# Phenotypic and molecular assessment of genetic structure and diversity in a panel of winged yam (*Dioscorea alata*) clones and cultivars

**DOI:** 10.1038/s41598-019-54761-3

**Published:** 2019-12-03

**Authors:** Paterne Agre, Flora Asibe, Kwabena Darkwa, Alex Edemodu, Guillaume Bauchet, Robert Asiedu, Patrick Adebola, Asrat Asfaw

**Affiliations:** 1 International Institute of Tropical Agriculture (IITA), Ibadan, Nigeria; 2 Department of Crop Protection, Federal University of Agriculture, Abeokuta, Nigeria; 3 Boyce Thompson Institute, Cornell University, Ithaca, New York USA; 4 International Institute of Tropical Agriculture (IITA), Abuja station, Nigeria; 5 Pan African University, Institute of Life and Earth Sciences, University of Ibadan, Ibadan, Nigeria

**Keywords:** Agricultural genetics, Plant breeding

## Abstract

A better understanding of the structure and extent of genetic variability in a breeding population of a crop is essential for translating genetic diversity to genetic gain. We assessed the nature and pattern of genetic variability and differentiation in a panel of 100 winged-yam (*Dioscorea alata*) accessions using 24 phenotypic traits and 6,918 single nucleotide polymorphism (SNP) markers. Multivariate analysis for phenotypic variability indicated that all phenotypic traits assessed were useful in discriminating the yam clones and cultivars. Cluster analysis based on phenotypic data distinguished two significant groups, while a corresponding analysis with SNP markers indicated three genetic groups. However, joint analysis for the phenotypic and genotypic data provided three clusters that could be useful for the identification of heterotic groups in the *D. alata* breeding program. Our analysis for phenotypic and molecular level diversity provided valuable information about overall diversity and variation in economically important traits useful for establishing crossing panels with contrasting traits of interest. The selection and hybridization of parental lines from the different heterotic groups identified would facilitate maximizing diversity and exploiting population heterosis in the *D. alata* breeding program.

## Introduction

Yam (*Dioscorea* spp.) is an important tuber crop supporting the livelihood of millions of people in the tropics and subtropics^1,2^. Globally, the average annual yam production is about 60 million tons with a gross value of ~14 billion US dollars. West Africa is the major yam growing area accounting for 94% of global yam production^3^. There are over 600 *Dioscorea* species of which ten are cultivated and are of economic importance^4^. The winged yam (*Dioscorea alata*), which is also called water yam or greater yam, is widely distributed in Africa and other tropical areas of the world^5^. *Dioscorea alata* is known for its vigorous plants with high tuber yield and plays a vital role in food security and income generation^6^. Different ecotypes are currently grown and used^7^.

Assessing genetic diversity present within a plant species is essential to identify genes controlling useful biological functions that can then be rationally used to develop new varieties^8^. The level and extent of genetic diversity in yam crop in general^9–12^ and winged yam specifically^13,14^ have been assessed using phenotypic attributes and low throughput molecular markers. Norman *et al*.^13^ successfully characterized 52 accessions of *D. alata* from Sierra Leone using 28 morphological descriptors. Anokye *et al*.^14^ dissected phenotypic diversity in a collection of *D. alata* from Ghana and Nigeria using morphological descriptors and grouped the accessions into distinct clusters independent of geographic origin. In addition to the phenotypic diversity, DNA-based marker systems using low throughput marker types^4,7,15,16^ have been applied to assess the genetic diversity of various *D. alata* populations. *Dioscorea alata* accessions from diverse geographical areas of West and central Africa and Puerto Rico were clustered into three genetic groups using AFLP markers irrespective of geographical origin^16^. Arnau *et al*.^7^ profiled 348 accessions using 24 microsatellite markers and reported wide genetic variation and significant structuring associated with geographic origin, ploidy levels, and morpho-agronomic characteristics.

Recent advances in sequencing techniques and their cost reduction has provided new insight into population structure and genetic variability in crops. Reference genomes are now available for both *D. rotundata* and *D. alata* (https://yambase.org/jbrowse_yambase/?data=data/json/) as well as a high-density genetic map of winged yam^2^. The availability of genomic resources in *D. alata* enables genome-wide study on genetic diversity with large datasets of high-density SNP markers from the next-generation sequencing platforms. Diversity Array Technology (DArT) characterized by high call rates and scoring reproducibility is among the sequencing techniques that are successfully applied in exploring genetic diversity and population genetic parameters of diploid root crops including cassava^17^ or polyploids such as potato^18^ but has not yet been applied to yam.

Previous diversity studies in yam have generally been conducted using either morphological or low throughput molecular markers. However, joint analysis of morphological and molecular information has the potential to provide in depth insight into population structure and genetic variability and it has been successfully applied in other crops^4,19–22^. The usefulness of this approach for genetic variability analysis in yam was demonstrated by Sartie *et al*.^4^. Employing a combination of data from 32 microsatellite markers and 24 phenotypic traits, *D. alata* and *D. dumetorum* which hitherto were categorized as sub-groups were clearly separated into distinct genetic groups^4^.

The objective of this study was to assess the genetic structure and diversity in a panel of *D. alata* clones and cultivars using phenotypic traits and SNP markers. These results will serve as a basis for a protocol to evaluate the genetic diversity and make an informed choice of parental lines to design new varieties in *D. alata*.

## Results

### Diversity and differentiation based on phenotypic traits

We performed principal component analysis to identify the most discriminative variables among the accessions. The first seven principal components cumulatively explained 76% of the total phenotypic variation (Table 1). The first principal component explained 25% of the total variation with the main contribution for this observed variation coming from eight variables. Principal component two accounted for 18% of the total variation with traits such as the number of medium and small tubers, number of stems, total tuber number per plant, the weight of medium and small tubers and length of medium and small tubers contributing much to this factor. Three variables, including tuber texture, tuber appearance, and tuber hairiness, were the qualitative traits that contributed much to the principal component three. The width of medium tubers contributed most to the variation explained by the fourth principal component. Two variables, senescence class, and tuber surface cracks contributed much to the variation explained by principal component 5. Yam anthracnose disease severity and tuber shape contributed most to the principal components 6 and 7, respectively. Based on the contribution of each of the measured traits to the most informative principal components, all the 24 variables were found to be relevant in discriminating the water yam accessions.

**Table 1:** Principal component analysis and variables contribution on each factor (Values in bold indicate the most relevant characters (>0.450) that contribute to the variation of the components).

Variables	PC.1	PC.2	PC.3	PC.4	PC.5	PC.6	PC.7
Senescence class	0.049	0.187	0.258	−0.232	**0.632**	0.302	−0.262
Tuber texture	0.226	−0.312	**0.644**	−0.323	0.266	0.229	0.077
Tuber shape	0.224	−0.080	0.203	−0.198	−0.416	0.395	**−0.552**
Tuber appearance	−0.015	0.352	−**0.618**	0.375	0.078	−0.127	−0.309
Tuber cracks	−0.258	−0.044	−0.103	0.163	**0.661**	−0.144	0.063
Number of big tubers	**0.887**	0.017	0.016	0.205	0.062	0.062	−0.015
Number of medium tubers	0.338	**0.718**	−0.370	−0.176	−0.040	0.076	0.144
Number of small tubers	−0.400	**0.705**	0.406	0.213	0.034	0.015	−0.106
Number of stems	0.337	**0.602**	0.249	0.194	0.058	−0.058	0.098
Stem girth	**0.504**	−0.292	0.029	0.286	−0.201	−0.081	0.380
Total tuber weight	**0.866**	0.296	0.061	0.083	0.055	−0.004	0.153
Total tuber number	0.151	**0.867**	0.267	0.205	0.056	0.010	−0.021
Weight of big tubers	**0.815**	−0.210	0.132	0.238	0.084	0.151	0.153
Weight of medium tubers	0.385	**0.516**	−0.384	−0.364	0.019	−0.009	0.181
Weight of small tubers	−0.361	**0.729**	0.398	0.262	−0.016	0.065	0.010
YAD	−0.461	−0.023	−0.305	0.316	0.000	**0.611**	0.286
YMV	**−0.633**	−0.130	−0.169	0.222	−0.062	0.534	0.103
Tuber hairiness	0.478	0.068	**0.540**	−0.292	−0.189	−0.008	0.171
Length of big tubers	**0.813**	−0.107	−0.040	0.307	−0.023	0.114	−0.140
Length of medium tubers	0.357	**0.613**	−0.331	−0.321	−0.141	0.080	−0.122
Length of small tubers	−0.314	**0.634**	0.205	0.159	−0.198	−0.001	−0.037
Width of big tubers	**0.805**	−0.168	0.000	0.333	0.032	0.194	−0.063
Width of medium tubers	0.126	0.370	−0.369	**−0.470**	0.108	0.257	0.279
Width of small tubers	**−0.614**	0.129	0.358	0.003	−0.177	−0.017	0.349
Eigenvalue	6.084	4.399	2.507	1.697	1.294	1.165	**1.106**
% variance	25.351	18.331	10.446	7.070	5.392	4.856	**4.610**
Cumulative variance (%)	25.351	43.682	54.128	61.198	66.590	71.446	**76.056**

YAD: Yam anthracnose disease; YMV: Yam mosaic Virus.

Correlation analysis revealed high and positive correlation between tuber yield components such as total tuber number (big, medium and small), the weight of the tuber (big, medium and small) and the length (big, medium and small) while negative correlations were observed between the tuber yield components and the two disease variables (Supplementary Fig. S1).

**Figure 1:**
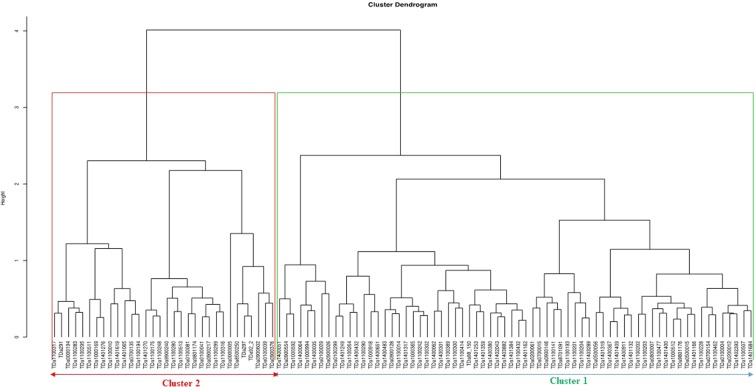
Hierarchical cluster dendrogram based on ‘Gower’ genetic similarity matrix of 24 phenotypic traits showing the grouping pattern of the *D. alata* accessions evaluated.

Assessment of phenotypic diversity using morphological attributes grouped the accessions into two distinct clusters (Fig. 1). Cluster one recorded the highest number of accessions (68), all of which were breeding lines. Accessions in cluster one had long tubers, high tuber yield and moderate tolerance to the yam mosaic virus (YMV) and to yam anthracnose diseases (YAD). Cluster two had 32 accessions, including the three farmer’s varieties (TDa291, TDa297, and TDa92_2). Accessions in this cluster were poor in tuber yield (small tubers) and susceptible to YAD and YMV.

### Genetic diversity parameters and population structure with SNP markers

Minor allele frequency varied from 0.05 to 0.5, with an average of 0.25. Expected heterozygosity varied from 0.10 to 0.50, with an average of 0.34. The observed heterozygosity ranged from 0.03 to 0.69, with an average of 0.38 across the SNP markers. Average polymorphism information content was 0.36. The SNP markers were unequally distributed across the 21 chromosomes, with the highest number observed on chromosome 5 and the lowest observed on chromosome 11 (Supplementary Fig. S2). Chromosome 5 presented the highest SNP density followed by chromosomes 4 and 8.

**Figure 2:**
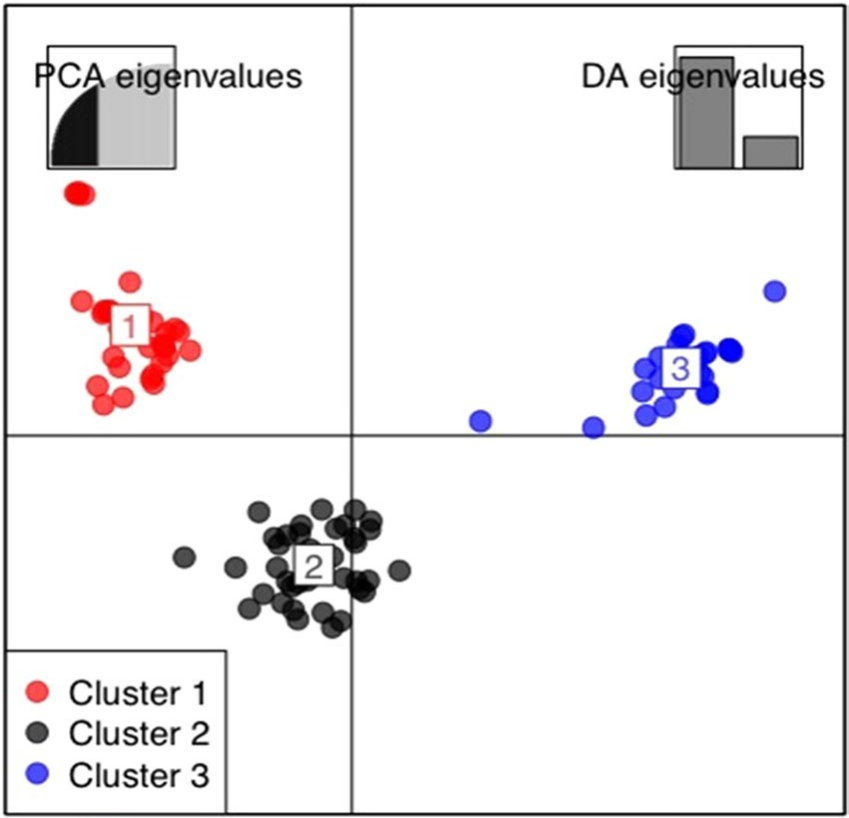
Discriminant analysis of principal components (DAPC) using 6918 SNP markers. The axes represent the first two Linear Discriminants (LD). Each colour represents a cluster, and each dot represents an individual. Numbers represent the different subpopulations identified by DAPC analysis.

Using the Bayesian information criterion (BIC) and the discriminant analysis of principal components (DAPC), a maximum of k = 3 was obtained (Supplementary Fig. S3) which corresponded to the number of clusters under the DAPC. Estimation of the cluster membership revealed that cluster two had the highest number of accessions (42) followed by cluster one with 33 accessions and cluster three with the smallest number of accessions (27) (Fig. 2). The genetic similarity among and within the three DAPC clusters showed variation for the fixation index (F_ST_) with the highest F_ST_ (0.13) obtained between accessions in cluster one and cluster three, while the lowest value was found between accessions of DAPC clusters one and two (F_ST_ = 0.06)_._ Assessment of the genetic diversity among accessions within each cluster revealed very low F_ST_ variability of −0.012, −0.011 and −0.014 for clusters 1, 2 and 3, respectively (Supplementary Fig. S4).

The genetic distance of 0.03 was observed between the intentional duplicate DNA samples of TDa1000512a and TDa1000512, while 0.02 was obtained between TDa1100242a and TDa1100242 (Supplementary Table S1). Hence, a threshold of 0 ≤ x ≤ 0.03 (where x is the genetic distance between two accessions) was set, and accessions with genetic distances lower than this threshold were considered as duplicates or closely related. The genetic distance varied from 0.02 to 0.83, and the highest value was observed between TDa291 and TDa0000005.

**Figure 3:**
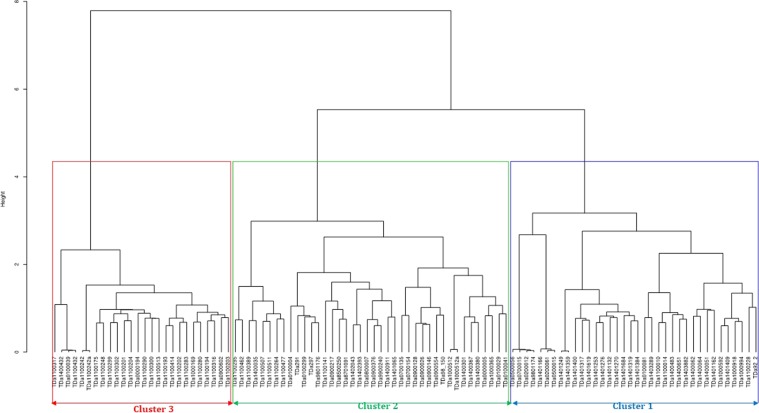
Hierarchical cluster dendrogram based on ward.D2 method showing the genetic relationships among the yam accessions based on a Jaccard genetic similarity matrix obtained from the 6918 SNP markers. In each group, accessions with high genetic similarity (near 0) were grouped in the same branch.

Based on the dissimilarity matrix generated from the SNP markers, the cophenetic coefficient correlation for the ward.D2 was 0.70, and the accessions categorized into three major clusters (Fig. 3). A total of 26 accessions were grouped into cluster one with genetic distance ranging from 0.02 to 0.75 (Supplementary Table S2). In this cluster, clones TDa0500056, TDa0200012, TDa0700015, and TDa98011174 shared most of alleles and hence they can be considered as closely related as shown through the hierarchical cluster. In the same cluster, TDa1401249 and TDa1401359 were found to have the lowest genetic distance (GD = 0.02) and can also be classified as duplicates or very closely related. Cluster two included 40 accessions, of which 38 are breeding lines and two landraces (TDa291 and TDa297). In this cluster, the lowest genetic distance (GD = 0.03) was observed between the TDa1000512a and TDa10000512 (intentional duplicate) while the highest genetic distance was observed between TDa291 (landrace) and TDa0000005 (breeding line). In cluster three, the genetic distance varied from 0.02 (TDa0100039 and TDa1100432) to 0.62 (TDa1100280 and TDa1100302). Apart from the intentional duplicate DNA TDa1100242 and TDa1100242a accessions like TDa1100432, TDa0100039 and TDa1400432 were found with very minimal genetic distances, hence can be considered as closely related.

**Figure 4:**
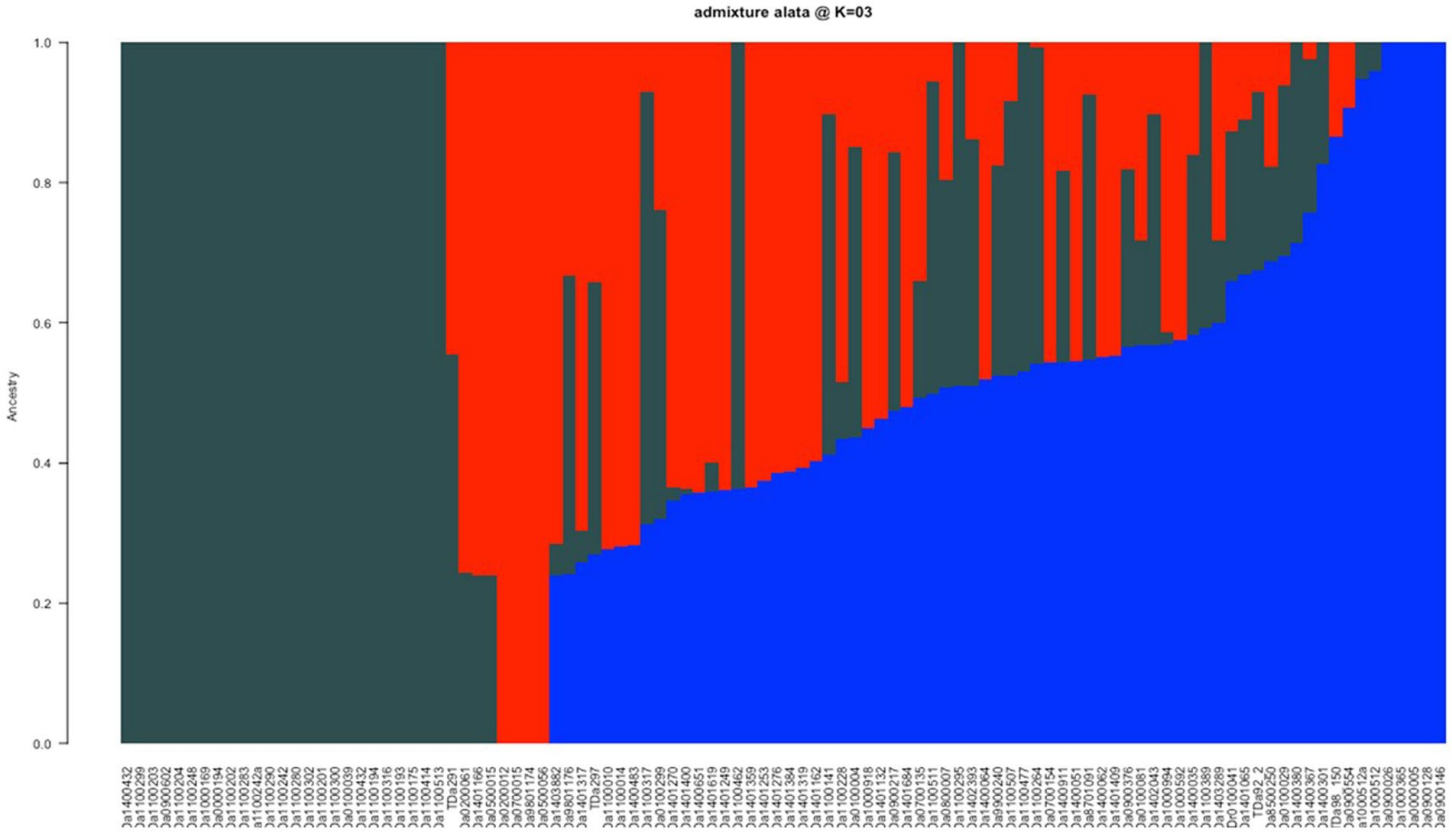
Admixture plot showing clustering of the *D. alata* accessions into three clusters based on the molecular data using Bayesian-based clustering analysis. A vertical bar represents each accession. The coloured sections in a bar indicate membership coefficients of the accessions in the different clusters. Identified subgroups are: cluster 1 (red), cluster 2 (black), and cluster 3 (blue).

Population structure analysis performed at K = 3 revealed high genetic diversity with a large proportion of admixture (individuals with multiple groups affiliation) (Fig. 4). Out of the 100 accessions, TDa0900026, TDa0200012, TDa0000005, TDa1100204, TDa0900128, TDa0700015, TDa9801174, TDa0500056, TDa0900146 were identified to be without admix and can be considered as pure lines. Also, the intentional duplicate genotype DNA TDa1000512 and TDa1000512a were found to have the same admix pattern (Fig. 4).

### Combined analysis of phenotypic and genotypic data

**Figure 5:**
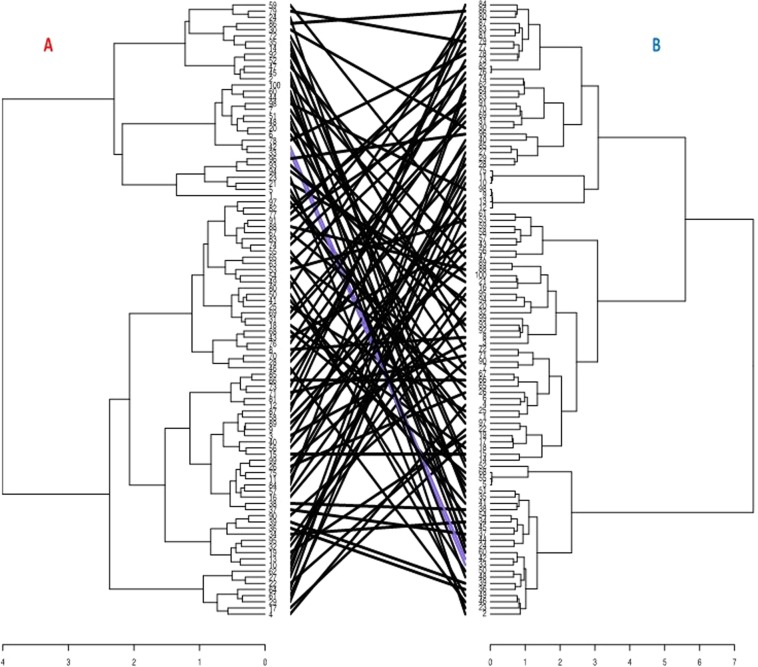
Comparison of hierarchical cluster dendrograms from phenotypic (**A**) and the genotypic (**B**) data. The black lines in between the two dendrograms represent mismatched accessions while the purple lines are accessions in the same position from phenotypic to the genotypic cluster.

Hierarchical cluster generated from the phenotypic information was compared to that originating from the genotypic data, and very few clones (2%) were identified to be clustered into the same position across the two hierarchical clusters (Fig. 5). We noticed grouping patterns and membership changes between phenotypic and molecular information. Forty percent of the total accessions evaluated maintained their groups both across the phenotypic and molecular clustering.

**Figure 6:**
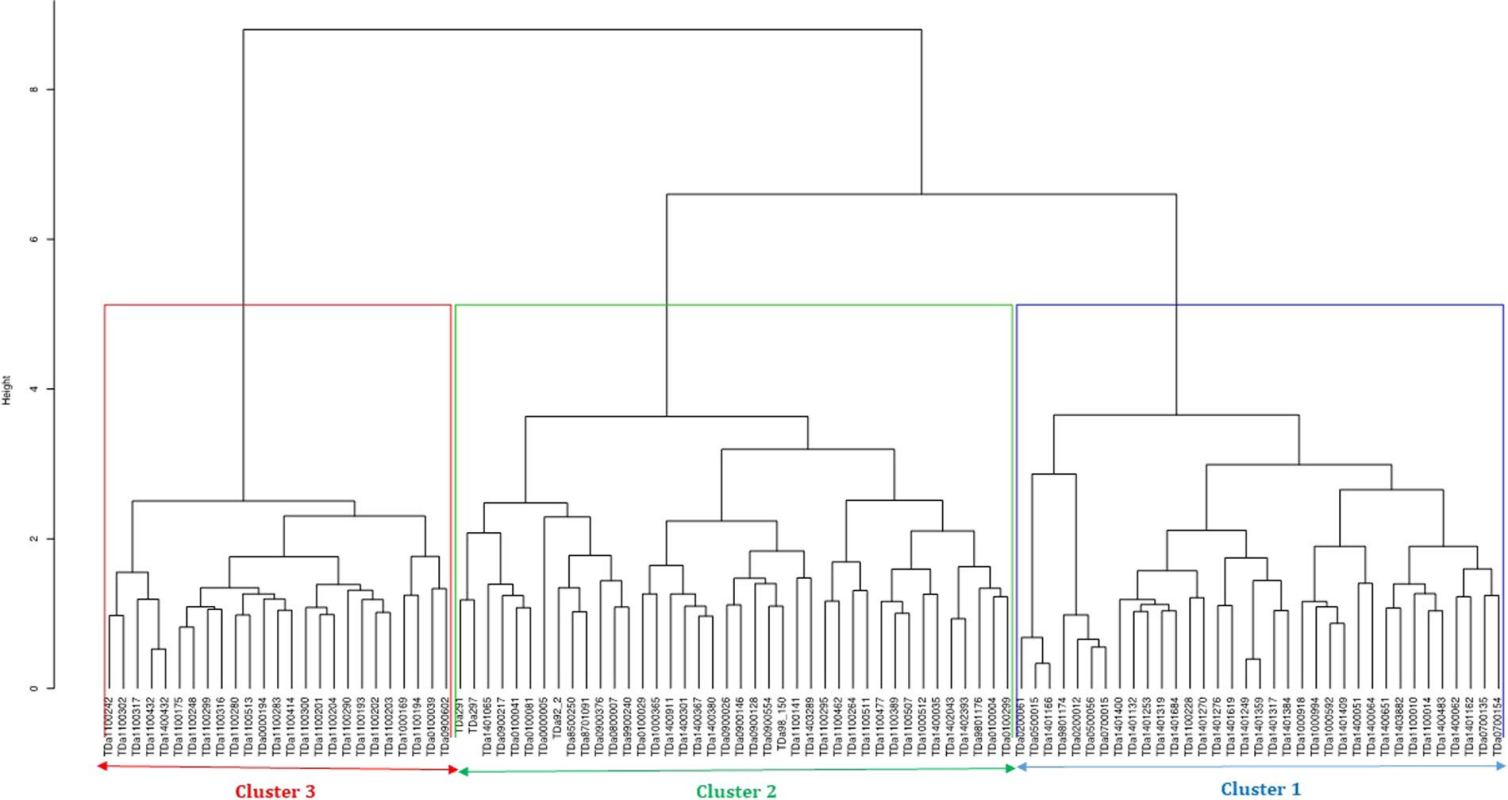
Hierarchical cluster based on the combined phenotypic and molecular data using Ward.D2 method.

Genetic diversity assessment using the combined phenotypic and molecular information revealed the presence of three well defined genetic groups in the current set of materials (Supplementary Figs. S5 and S6). Cluster one is composed of 35 accessions (Fig. 6), all of which are breeding lines. Accessions in this group mainly originated from bi-parental crossing between TDa0500015 (female parent) and TDa0200012 (male parent) (Supplementary Table S3). Thirty-seven breeding lines and three landraces were grouped in cluster two. A total of 25 accessions were grouped in the third cluster. Seventy-two percent of the clones in this cluster originated from open pollination with TDa00000194 as the female plant.

Discriminant analysis carried out on the three clusters emanating from the combined matrix showed a variation of both phenotypic traits and genotypic parameters (Table 2, Supplementary Fig. S6). Accessions categorized into cluster one have good tuber yield traits such as many tubers (long and large) without cracks (Table 2). Accessions grouped into cluster two expressed an average tuber yield but with rough tuber skin characteristics. The 25 accessions identified in cluster three were mainly characterized by less preferred tuber yield attributes such as small tuber weight, size, and length (Table 2). Concerning the genotypic parameters, polymorphic information content (PIC) varied considerably across the three groups while slight variation was observed for the rest of the parameters such as observed heterozygosity, expected heterozygosity (HE), minor allele frequency and major allele frequency (Table 2).

**Table 2:** Phenotypic traits variation and diversity indices statistics of the three clusters obtained from the combined phenotypic and genotypic matrices.

Variables	Traits	Cluster 1		Cluster 2		Cluster 3	
		Average	STD	Average	STD	Average	STD
Phenotypic	Senescence class	4.05	0.34	4.14	0.25	4.07	0.49
	Tuber texture	1.54	0.28	1.66	0.22	1.37	0.25
	Tuber shape	2.92	0.21	2.88	0.33	2.76	0.22
	Tuber appearance	1.86	0.19	1.83	0.22	2.00	0.21
	Tuber cracks	0.13	0.16	0.20	0.21	0.25	0.22
	Number of big tubers	1.25	0.46	1.00	0.58	0.77	0.43
	Number of medium tubers	1.52	0.40	1.38	0.53	1.42	0.44
	Number of small tubers	2.09	1.30	2.03	0.88	2.32	1.12
	Number of stems	1.48	0.29	1.35	0.25	1.32	0.21
	Stem girth	4.23	0.34	4.02	0.34	3.98	0.36
	Total tuber Weight	3.37	0.85	2.74	0.94	2.41	0.73
	Total tuber number	4.55	1.38	4.08	1.11	4.05	0.98
	Weight of big tubers	2.16	0.94	1.80	1.09	1.37	0.72
	Weight of medium tubers	1.19	0.32	1.07	0.35	1.10	0.43
	Weight of small tubers	0.54	0.28	0.53	0.22	0.61	0.25
	Yam anthracnose disease	2.44	0.11	2.50	0.12	2.63	0.15
	Yam mosaic virus	2.25	0.10	2.36	0.18	2.45	0.17
	Tuber hairiness	2.39	0.22	2.34	0.25	2.18	0.16
	Length of big tubers	23.31	5.98	19.25	7.67	17.39	8.01
	Length of medium tubers	21.00	2.63	17.76	4.34	19.62	4.16
	Length of small tubers	13.42	2.72	13.02	3.30	13.84	2.39
	Width of big tubers	27.65	4.67	23.15	9.22	20.66	9.04
	Width of medium tubers	22.04	3.11	21.47	4.57	21.40	3.59
	Width of small tubers	14.31	2.72	15.16	3.45	15.25	2.52
Genotypic	Minor Allele frequency	0.24	0.25	0.22			
	Major allele frequency	0.76	0.75	0.78			
	Observed heterozygosity	0.39	0.37	0.38			
	Expected heterozygosity	0.32	0.34	0.29			
	Polymorphism information content	0.25	0.27	0.23			

Big tuber (tuber with weight ≥1 kg), medium tuber (Tuber with weight ≥0.5 kg but less than 1 kg) small tuber (Tuber with weight less than 0.5 kg); STD (Standard deviation).

Dissimilarity matrix generated from the phenotypic data was compared with that obtained from the molecular data, and very low correlation (r = 0.097) was observed between the two matrices (Supplementary Fig. S7). High correlation values were detected between the joint matrix of combined distances and the genotypic and phenotypic information, with a Mantel test showing high (r = 0.84) and moderate (r = 0.53) values for phenotypic and genotypic data, respectively (Supplementary Fig. S7).

## Discussion

This study dissected the level and extent of genetic diversity in a panel of 100 *D. alata* clones and varieties routinely utilized in yam breeding program at IITA using both phenotypic and molecular markers. The results of our multivariate analysis with phenotypic traits indicated substantial diversity in the current set of *D. alata* accessions with all the measured traits having relevance for characterization of yam germplasm. However, a subset of traits including number of tubers, tuber weight, tuber length and width, stem diameter, number of stems and yam anthracnose disease had very high contributions to the principal components and could be used to efficiently assess diversity in *D. alata* collections. Several authors have also reported the importance of phenotypic traits in unravelling the diversity and differentiation in yam genotypes^13,14^. Phenotypic characterization is very relevant to define germplasm accessions or breeding lines and serves as meaningful criteria for selecting materials with desirable traits for breeding endeavors.

Various types of molecular markers ranging from dominant to codominant are available to estimate and assess the level of genetic diversity. This study also used 6918 SNP markers from Diversity Array Technology (DArT) platform to elucidate the genetic diversity and population structure of the *D. alata* accessions. The usefulness of the DArT technology for genetic diversity analysis has been well established for many crops^23–25^. The three complementary approaches implemented here, namely DAPC, hierarchical cluster, and admixture ancestry to define the optimal number of groups all showed the presence of three genetic groups among the 100 winged yam accessions. The high agreement in the membership of the hierarchical clusters and DAPC groups found in this study was also observed by Wang *et al*.^26^ and Fatokun *et al*.^27^ in their study of genetic diversity and population structure of rice and cowpea accessions, respectively. The observed genetic differentiation among the studied materials was further confirmed by the high fixation index (Fst) value among accessions between clusters and low F_ST_ value among accessions within clusters. This indicated high connectivity among accessions within clusters and low connectivity among accessions between clusters as also reported in Cowpea^27^ and Cassava^28^ using DAPC analysis with SNP markers from genotyping by sequencing. The high cophenetic coefficient correlation (>0.70) observed here for the hierarchical cluster constructed based on the molecular data indicates the reliability of this approach to summarize the information of dissimilarity matrices^21^.

The high proportion of admixture observed in our study is an indication of gene flow in the population of *D. alata*. Many of the accessions in our study are breeding lines emanating from the hybridization of genotypes of diverse genetic backgrounds. Such hybridization has enhanced the genetic diversity as rightly asserted by Arnau *et al*.^7^. The few accessions identified with no admixture can be utilized to develop bi-parental crosses for QTL identification while focusing on the traits of interest in the breeding program.

The low correlation observed between the genotypic and phenotypic distance matrices observed in this study was also reported in sweet potatoes^21^, Cupuassu^22^, Jatropha^29^ and yellow passion fruit^30^, and in agreement with the inconsistencies observed between the clusters generated from the phenotypic and the molecular diversity analyses. The negligible association observed between the phenotypic and genotypic data could be because the variation in phenotypic traits may be as a result of one or few mutations and therefore often not concordant with the overall genetic distance between two accessions. Consequently, to assess the amount (and differentiation among e.g. geographical areas or other relevant entities) of total anonymous variability, DNA markers are often more efficient since they rely on numerous mutations along the entire genome. This low correlation between phenotypic and molecular diversity matrices could also be because the variation often detected by molecular markers is commonly of the non-adaptive type and hence not liable to natural and or artificial selection, unlike phenotypic characters which are usually subject to natural and artificial selection^31^. Additionally, molecular markers have been assumed to be selectively neutral, whereas the portions of the genome associated with phenotypic expression are subject to selection under environmental influence^32,33^. Several authors, therefore, suggest that the best way to identify divergence among accessions is the combined use of molecular and phenotypic data as these tools are complementary^4,20,31,32^. The low correlation between morphological and molecular data in the *D. alata* accessions generally suggests that the two data types are appropriate for a combined use, which can deepen understanding and discriminate the genotypes better due to the non-overlapping information.

The genetic diversity and differentiation in the population were also assessed using a joint dissimilarity matrix of the phenotypic and molecular marker data resulting in three distinct clusters. The high and moderate correlation observed between the combined dissimilarity matrix and the genotypic and phenotypic dissimilarity matrices, respectively, is an indication that genetic diversity analysis based on the joint matrix is a very valuable tool to cumulatively and reliably allocate genotypic and phenotypic information. The usefulness of the combined dissimilarity matrix of morphological and molecular data in deciphering the genetic variability and differentiation among accessions in this study corroborate the findings of Sartie *et al*.^4^, Andrade *et al*.^21^, Alves *et al*.^22^, Reis *et al*.^30^ and Alves *et al*.^32^. By analyzing morphological and molecular data together, Alves *et al*.^32^ found that the average dissimilarity among physic nut accessions improved by 156% and 64% with respect to the original mean phenotypic and molecular dissimilarities, respectively. Similarly, Martins *et al*.^34^ and Bosetti*et al*.^35^ observed a marked increase in the sampling intensity of the genetic diversity by combining phenotypic and molecular data. This could be as a result of the combination of non-overlapping information into one data set^32^.

In conclusion, our analysis of the diversity in the *D. alata* clones and cultivars using genotypic, phenotypic, and combined genotypic/phenotypic distances revealed ample genetic variation in the population. This diversity can be explored for *D. alata* improvement and genetic resource conservation. As a result of the inconsistencies and reshuffling of cluster membership observed across the phenotypic and genotypic characterizations, the combined matrix could be employed for genetic diversity analysis if one desires to use both the phenotypic and genotypic information as this approach maintains high correlation with both matrices and gives a cumulative overview of the total diversity in the population. Future analysis to estimate the combining ability of the accessions in the three clusters identified by the combined distance will enhance the partitioning of the accessions into distinct heterotic groups. The selection and hybridization of parental lines from the three genetic groups identified by the combined morphological and molecular marker analysis is expected to maximize genetic diversity and heterosis in yam breeding program.

## Methods

### Plant material

A panel of 100 winged yam accessions obtained from the IITA yam breeding program was used for this study. These accessions comprised of 97 breeding lines emanating from full-sib and half-sib breeding and three landrace cultivars (Supplementary Table S3).

### Phenotypic evaluation

The accessions were planted in three locations in Nigeria: Ibadan (N7°40′19.62, E3°91′73.13) and Ikenne (N6°51′00.873, E3°41′48.528) in the south-west, and Ubiaja (N6°39′48.772″, E6°20′29.533) in the south-central part of the country. The experiment was laid out in a simple lattice design with three plants per genotype. Twenty-four phenotypic variables were recorded according to the yam crop ontology^36^. The different variables, collection period, and methods of assessment are summarized in Supplementary Table S4.

### Genotyping

One gram of young, healthy and fully expanded leaves from fully grown plants in the field were collected per genotype. The leaf samples were immediately put in Ziploc bags and kept on ice before transferring to −80 °C for freeze drying. Deoxyribonucleic acid (DNA) was extracted from the leaf samples using the CTAB procedure with slight modification^37^. DNA quality was estimated for each sample based on electrophoresis in an agarose gel.

DArT genotyping was performed using the methodology described by Sansaloni *et al*.^38^ with slight modification. For the sequencing-based DArT genotyping, complexity reduction methods optimised for yam at DArT was used: PstI_ad/TaqI/HpaII_ad with TaqI restriction enzyme used to eliminate a subset of PstI –HpaII. PstI-site specific adapter was tagged with 102 different barcodes enabling encoding a plate of DNA samples to run within a single lane on an Illumina GAIIx. After the sequencing, FASTQ files generated by DArT were aligned against the yam genome reference available on (https://drive.google.com/drive/folders/0B54dLJoyUZ5SUDY5TzV2eGFWaWs) and hapmap file was generated by DArT.

### Multivariate analysis of phenotypic data and hierarchical cluster construction

Analysis of variance was performed to determine differences among the accessions for the various traits across the three locations using the SAS version 9.4^39^ according to the model:

$${Y}_{ijkl}=\mu +B{(E)}_{j(i)}+{G}_{k}+G{E}_{ij}+{e}_{ijkl}$$

where: Y = trait, µ = grand mean, E = environment effect (location), B(E) = Block effect in environment, G = genotype effect, GE = genotype by environment interaction, e = error.

The LSmeans from the genotype by environment analysis was used for principal component analysis in the FactorMiner and missMDA R packages^40^. The optimal number of factors to be retained was determined using the dimdesc function in R, and the contribution of each trait was determined using the principle of Peres-Nero *et al*.^41^. A dissimilarity matrix was generated using the Gower method implemented in cluster and graphics R packages^42^. Count variables (such as number of tubers, numbers of stems) were transformed using log ratio function in R. The final hierarchical cluster was then performed using the ward.D2 method in cluster R package^42^. Correlation among the different phenotypic variables was performed using the R software and result was displayed as heatmap.

### Analysis of molecular data

To evaluate the sequencing error and reliability of the Diversity Array Technology for yam, two intentionally duplicated DNA samples were included. The Duplicated DNA was used to define the threshold for duplicate identification in the sample. The HapMap file received from the DArT platform was converted into a variant call format (VCF). A total of 22,140 SNP markers were identified from the raw data and after filtering with VCFtools^43^ for MAF (0.05), no missing data, depth (>5), Genotype Quality (GQ = 20), maximum and minimum allele = 2 and no indels, a total of 6,918 SNP markers were retained for further analysis.

Summary statistics including major and minor allele frequencies, heterozygosity both for SNP markers and genotypes were assessed using TASSEL software version 5.2.51^44^ and the plink command–freq and –hardy^45^. A population structure analysis was performed based on Admixture^46^ through the Bayesian Information Criterion (BIC). The optimal number of clusters was inferred using k-means analysis after varying the possible number of clusters from 2 to 40. A discriminant analysis of principal components (DAPC) was carried out on the identified clusters using the first 40 principal components in the adegenet R package^47^. Membership probabilities of the individuals for the different groups were estimated using a cut-off value of 40% suggested through the DAPC. Pairwise population differentiation statistics (F_ST_) was calculated using VCFtools^43^ to estimate the genetic distance and the relationships among the different DAPC groups formed. A pairwise genetic distance matrix was calculated using the Jaccard method implemented in the phylentropy R package^48^. A Ward’s minimum variance hierarchical cluster dendrogram was then generated from the genetic distance matrix using the analyses of phylogenetics and evolution (ape) R package^49,50^.

### Joint analysis of phenotypic and molecular data

Genetic groups were defined using a combination of the phenotypic and genotypic dissimilarity matrices. This joint matrix was generated by the summation of the genotypic and phenotypic dissimilarity matrices. A Mantel test was performed for analysis of correspondence between the dissimilarity matrices obtained from the phenotypic, genotypic and joint distance matrices using the Monte-Carlo method with 9999 permutations for estimation of significance. The hierarchical clusters generated from the phenotypic and genotypic data sets were compared using the viridis R package^51^ and the similarity of the two dendrograms assessed using the tanglegram function implemented in the dendextend R package^52^.

## Supplementary information

Supplementary Information

Pairwise genetic distance between the 100 D. alata accessions studied

Jaccard dissimilarity matrix of the three genetic groups formed through the hierarchical clustering method

List of accessions, pedigree information and categorisation into different clusters by DAPC and Hierarchical clustering
