# Supplementary material for: Phenotypic and molecular assessment of genetic structure and diversity in a panel of winged yam (Dioscorea alata) clones and cultivars
Source: Sci Rep. 2019 Dec 3;9:18221. doi: 10.1038/s41598-019-54761-3 (PMC6890776; doi:10.1038/s41598-019-54761-3)
Supplement: Supplementary file 1 — Supplementary Information [file 41598_2019_54761_MOESM1_ESM.pdf]

Manuscript title: Phenotypic and molecular assessment of genetic structure and diversity in a panel of winged yam (*Dioscorea alata*) clones and cultivars

Authors: Paterne Agre; Flora Asibe; Kwabena Darkwa; Alex Edemodu; Guillaume Bauchet; Robert Asiedu; Patrick Adebola & Asrat Asfaw

Supplementary Fig S1: Heatmap showing the correlation among the 24 phenotypic variables; Sen: Senescence class; Tu_tex: Tuber texture; Tu_sh: Tuber shape; Tu_ap: Tuber appearance: Tu_cr: Tuber cracks; No_big: Number of big tubers; No_med: Number of medium tubers; No_sma: Number of small tubers; Stem_no: Number of stems; Stem_girth: Stem girth; Total_tuber_WT: Total tuber weight; Total_tuber_no: Total tuber number; WT_big: Weight of big tubers; WT_med: Weight of medium tubers; WT_sma: Weight of small tubers; YAD: Yam anthracnose disease; YMV: Yam mosaic virus; hairs: Tuber hairiness; Len_big: Length of big tubers; Len_med: Length of medium tubers; Len_sma: Length of small tubers; Wid_big: Width of big tubers; Wid_med: Width of medium tubers; Wid_sma: Width of small tubers.

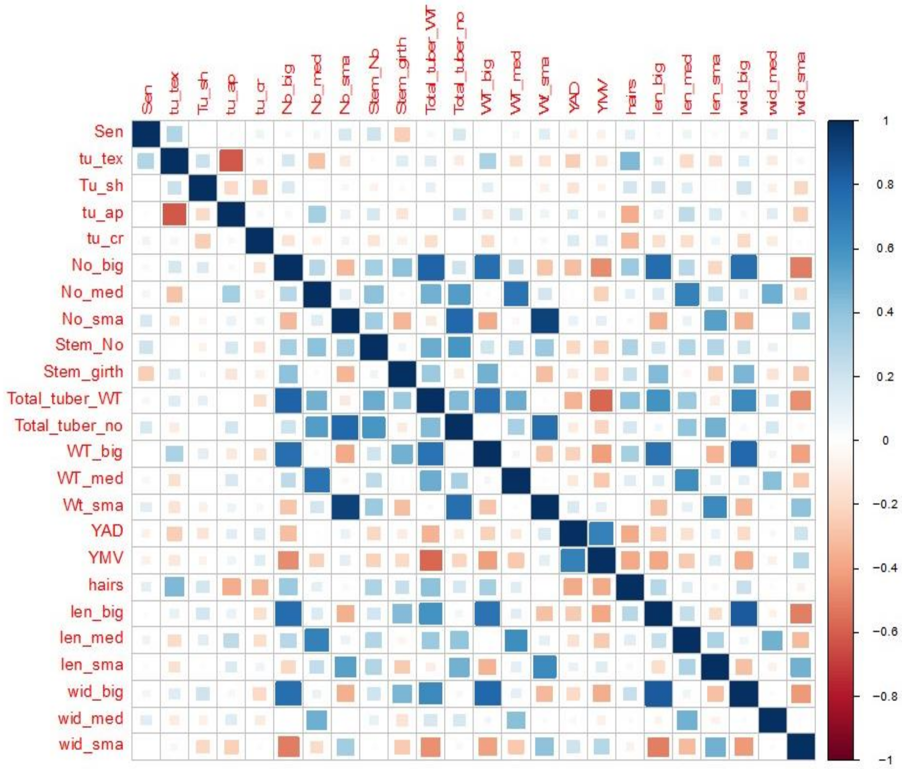

Supplementary Fig S2: SNP distribution on the 21 chromosomes (A): The horizontal axis depicts the chromosome length in Mb; the 0 ~ 747 legend insert shows the SNP density per chromosome; (B): Variation in the number of SNP markers across the chromosomes.

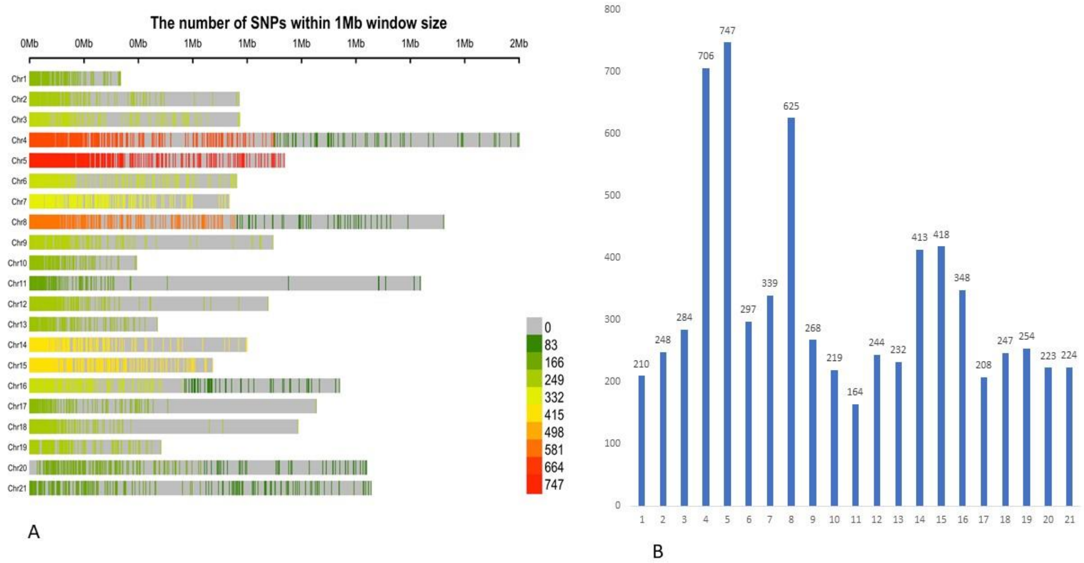

Supplementary Fig S3: Bayesian information criterion plotted against the number of clusters, the chosen number of clusters is the minimum number of clusters after which the BIC increases or decreases by a negligible amount.

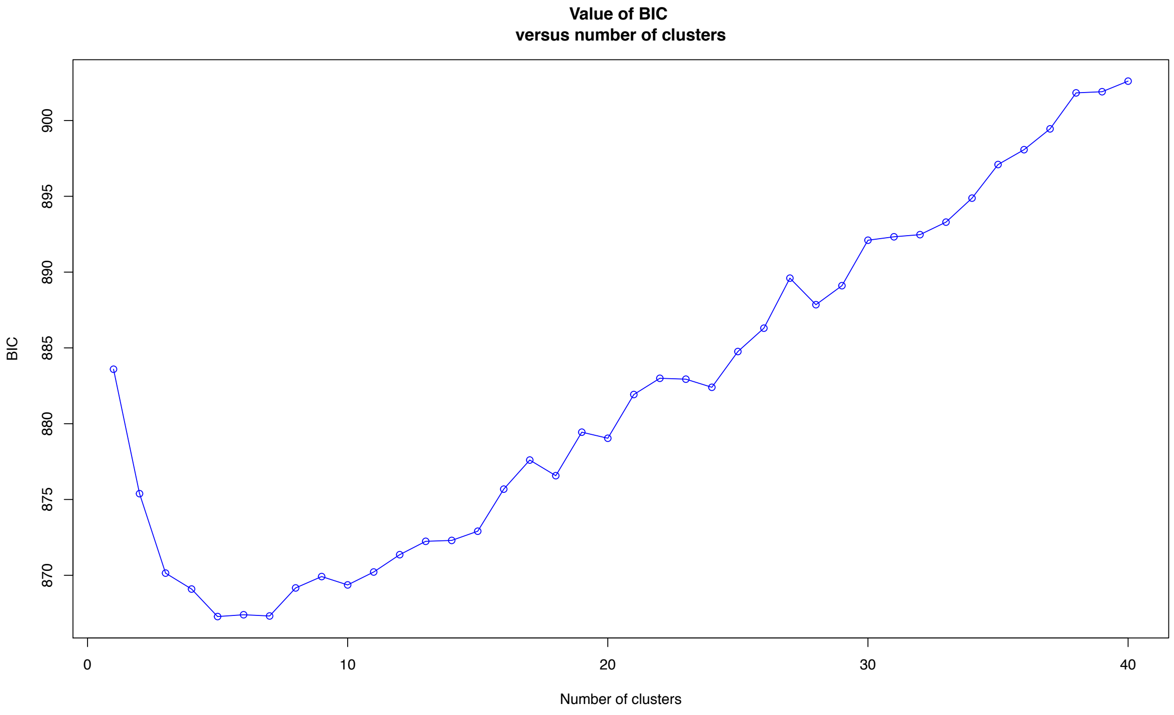

Supplementary Fig S4: Relationship within and among accessions of DAPC groups. Figure showing the divergence between the three DAPC groups and the variation occurring within groups

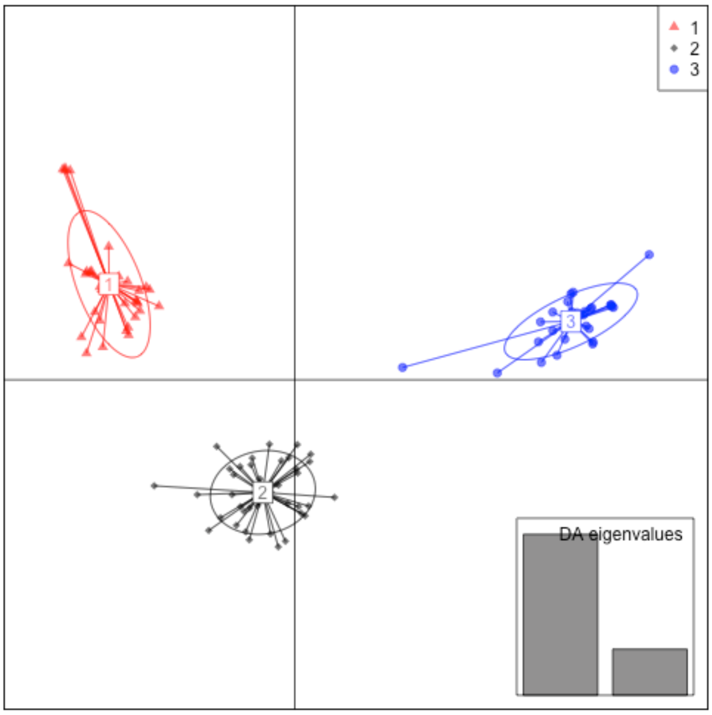

Supplementary Fig S5: Optimal number of clusters identified by using the combined genotypic and phenotypic data.

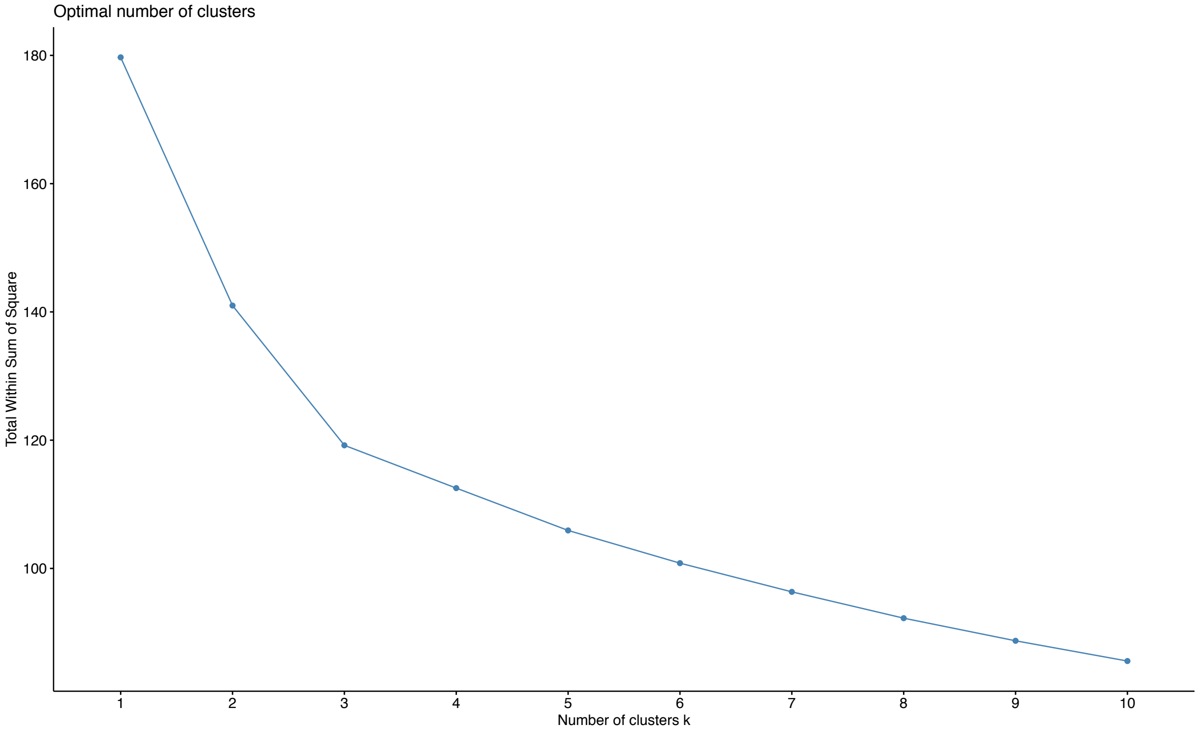

Supplementary Fig S6: Discriminant analysis showing the relationship among the 100 *Dioscorea alata* accessions assessed using the combined phenotypic and molecular data. The dots and serial numbers represent distinct accessions. The blue colour represents accessions in cluster 1, the pink represents accessions in cluster 2, and the green colour stands for the accessions in the third cluster.

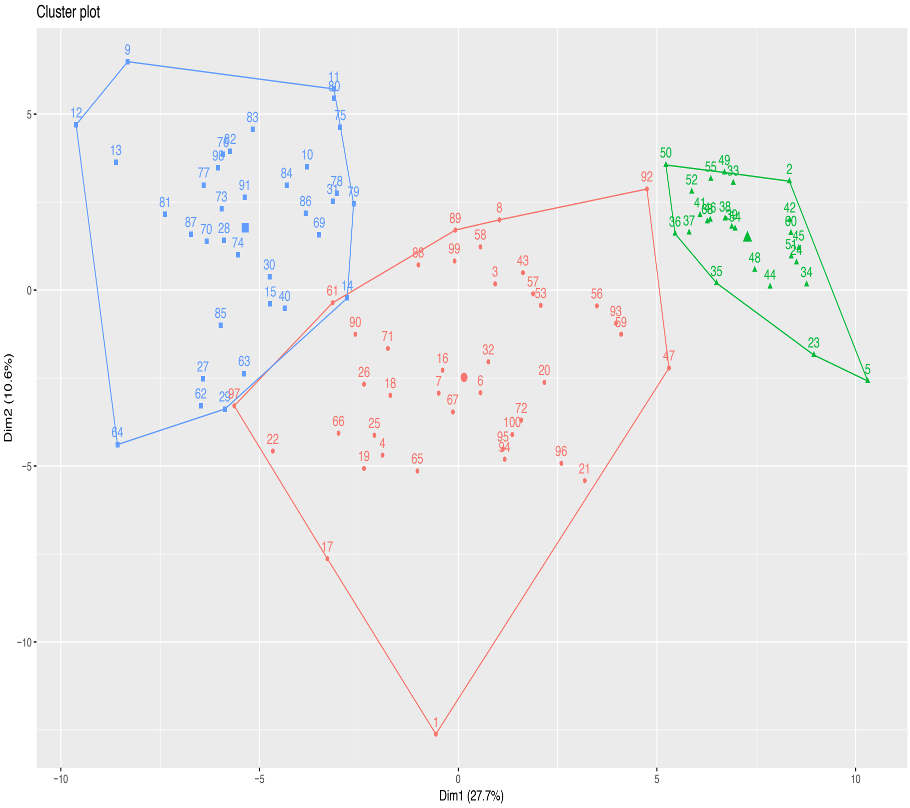

Supplementary Fig S7: Mantel correlation test performed between the different dissimilarity matrices alongside the combined matrix. Sim = similarity coefficient, beacon = Mantel threshold indicating the correlation.

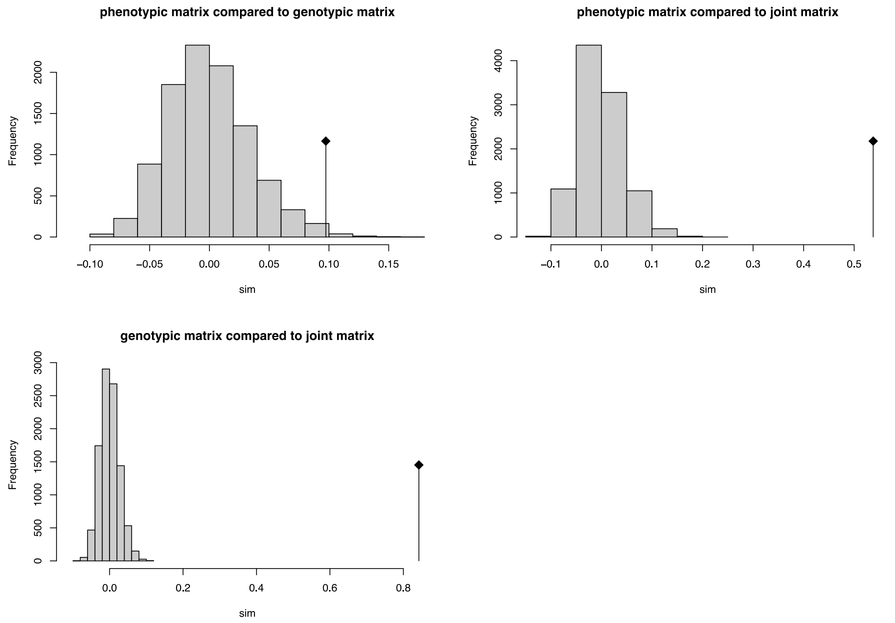

Supplementary table S4: Traits recorded, period of assessment and data method of evaluation

SN	Phenotypic traits	Collection period	Method
1	Yam Anthracnose Disease	2 months after planting and 15 days subsequently	Visual assessment on a scale of 1 to 5, where 1 = no visible sign of anthracnose disease, 2 = few symptoms covering 1 to 25% of the plant, 3 = symptoms covering 26 to 50% of affected plant, 4 = symptoms covering more than 51% of the plant and 5 = severe necrosis and ultimately plant death.
2	Yam Mosaic Virus Disease	2 months after planting and 15days subsequently	Visual examination on a scale of 1-5, where 1 = no visible symptom and virus negative, 2 = mosaic on most leaves and symptom progression with time, 3 = mild symptoms on few leaves without change in leaf shape (distortion), 4 = severe mosaic on most leaves with visible leaf distortion, 5 = severe mosaic (bleaching), sever leaf distortion and stunting.
3	Stem girth	Four months after planting	stem diameter was measured at 15cm from the base of the plant using the Vernier caliper
4	Stem Number	Four months after planting	Counting the number of stems per plant.
5	Number of big tuber	harvest time	Counting of tubers weighing 1kg and above
6	Number of Medium tuber	harvest time	Counting of tubers weighing 0.5kg and above but less than 1kg
7	Number of Small Tuber	harvest time	Counting of tubers weighing less than 0.5kg
8	Weight of Big tuber	harvest time	Weight of big tubers was measured using a sensitive electronic weighing scale (AND HV-60KC, A&D company Ltd, Korea).
9	Weight of medium tuber	harvest time	Weight of tubers medium sized tubers was measured using sensitive electronic scale
10	Weight of small tuber	harvest time	Weight of tubers small tubers was measured with a sensitive electronic weighing scale

11	Length of big tuber	harvest time	Measured using a flexible plastic measuring tape
12	Length of medium Tuber	harvest time	measured using a flexible plastic measuring tape
13	Length of small tuber	harvest time	measured using a flexible plastic measuring tape
14	Width of big tuber	harvest time	measured using a flexible plastic measuring tape
15	Width of medium tuber	harvest time	measured using a flexible plastic measuring tape
16	Width of small tuber	harvest time	measured using a flexible plastic measuring tape
17	Tuber Hairiness	harvest time	Visual assessment of the presence of hairs using a scale of 0, to 3, where 0 = No roots, 2 = few roots and 3 = many roots.
18	Tuber appearance	harvest time	Visual evaluation of tuber appearance using a scale of 1-3, where 1= Thorny, 2 = hairy, 3 = smooth.
19	Tuber cracks	harvest time	Visual assessment using a scale of 0, 1, 3 where 0 = absent, 1 = few, 3 = many.
20	Total tuber number	harvest time	Count of all the tubers harvested per plot
21	Total tuber weight	harvest time	Weight of all the tubers (big, medium and small) harvested per plot
22	Tuber texture	4 months	Tubers were evaluated using a scale of scale of 1-2, where 1= smooth and 2 = rough
23	Senescence class	6 months	Using the scale of 1to 9, where 1 = Very late, all the plants in a plot still show green foliage, 3 = Late, 75% of plants in a plot with still green foliage but few plants, 5 = Medium,50% of the plants were still green or on the onset of senescence, 7 = Early, plants have senescent foliage (75% of the plants in a plot with 50% leaves showing sign of yellowing but the vines still green), 9 = Very early, plants are completely senescent.
24	Tuber shape	harvest time	Determination of tuber predominant shape by visual assessment using a 1-4 scale where 1= Spherical, 2= Oval, 3= Cylindrical and 4= Irregular
